# Necrotizing funisitis and calcification of umbilical vein: case report and review

**DOI:** 10.1186/s12884-021-03743-3

**Published:** 2021-04-12

**Authors:** Wendi Wang, Miao Zhang, Liyun Gong, Qingqing Wu

**Affiliations:** 1 Department of Ultrasound, Beijing Obstetrics and Gynecology Hospital, Capital Medical University, 251 Yaojiayuan Road, Beijing, 100026 China; 2 Department of Ultrasound, Beijing Chaoyang Hospital, Capital Medical University, Beijing, China; 3 Department of Obstetrics and Gynecology, Beijing Chaoyang Hospital, Capital Medical University, Beijing, China

**Keywords:** Necrotizing funisitis, Intrauterine infection, Prenatal diagnosis, Ultrasonography, Umbilical vein calcification

## Abstract

**Background:**

Necrotising funisitis (NF) is a rare, chronic stage of funisitis, a severe inflammation of the umbilical cord and an important risk factor for fetal adverse outcomes. NF is characterized by yellow-white bands running parallel to the umbilical blood vessels. These bands consist of inflammatory cells, necrotic debris, and calcium deposits. Calcification is visible in ultrasonography, which makes it possible to suspect NF when umbilical vascular wall calcification is detected by prenatal ultrasonography.

**Case presentation:**

Ultrasonography revealed calcification of the umbilical venous wall in an expectant 31-year-old woman who was gravida 1, para 0. The woman required emergency cesarean section because of fetal distress and suspected umbilical cord torsion at 31 weeks gestation. The root of the umbilical cord was quite fragile and broke during the operation. The pathological results on the placenta showed histologic chorioamnionitis and NF. The infant was diagnosed to have neonatal sepsis and acidosis after delivery but was discharged without severe complications after a one-month hospitalization that included antibiotic and supportive therapy.

**Conclusion:**

NF is a rare and severe inflammation of the umbilical cord. Umbilical vascular wall calcification discovered in prenatal ultrasonography is diagnostically helpful.

**Supplementary Information:**

The online version contains supplementary material available at 10.1186/s12884-021-03743-3.

## Background

NF is a chronic and severe inflammation of the umbilical cord characterized by yellow-white bands paralleling the umbilical blood vessels. The bands consist of inflammatory cells, necrotic debris, and calcium deposits. NF increases the risk of fetal distress, intrauterine death, fetal growth restriction (FGR) and preterm labor [1, 2]. Jacques et al. [3] reported 45 cases of NF identified by placental histological examination with calcifications as the result of longstanding inflammation. Calcification of the umbilical vascular wall found by prenatal ultrasonography may indicate NF. There is little medical literature discussing the relevant ultrasonic findings of NF because the cases are rare and umbilical cord manifestations are easily ignored by ultrasound practitioners. Here, we describe a rare case presentation of NF with significant ultrasonic features and give a general review of NF.

## Case presentation

**Fig. 1:**
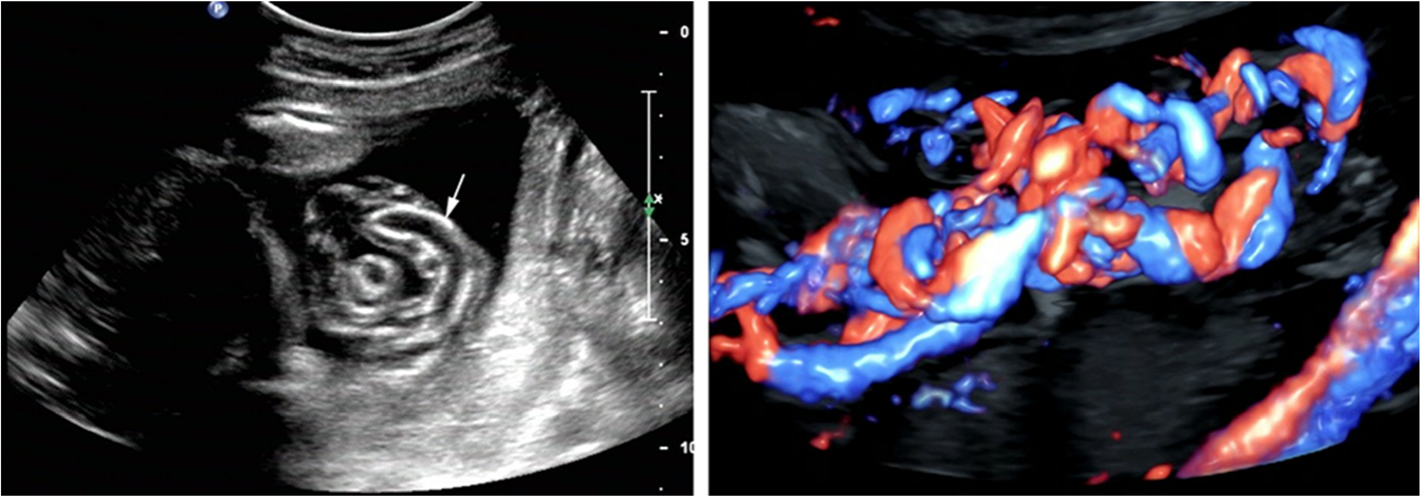
Ultrasonography image shows thickened and calcified umbilical vein wall (arrow) at 28 weeks of gestation (left). Three-dimensional color Doppler shows that the umbilical cord is stacked into a mass at 30 weeks of gestation (right)

A 31-year-old woman, gravida 1, para 0, underwent a routine antenatal examination in the hospital. During the pregnancy, subclinical hypothyroidism and gestational diabetes were diagnosed and the woman received appropriate treatment for these conditions. Minor vaginal bleeding had occurred at 25 weeks of gestation and ultrasonography found no abnormality. In the detailed ultrasonography examination at 28 weeks, the umbilical venous walls were thickened, with hyperechogenicity and calcification (Fig. 1 left). There were no unusual infective symptoms or signs such as fever, abdominal pain or foul-smelling vaginal discharge. The maternal white blood cell count and serum C-reactive protein (CRP) were all normal and the screenings for TORCH (toxoplasmosis, rubella, cytomegalovirus, and Herpes simplex virus) serology were negative.

**Fig. 2:**
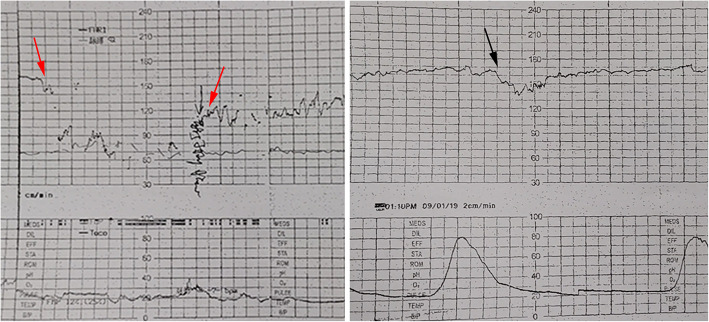
Fetal heart rate monitoring shows a spontaneous prolonged deceleration and the red arrows show the onset and end of fetal heart rate deceleration (left). Oxytocin challenge test shows a late deceleration (right)

**Fig. 3:**
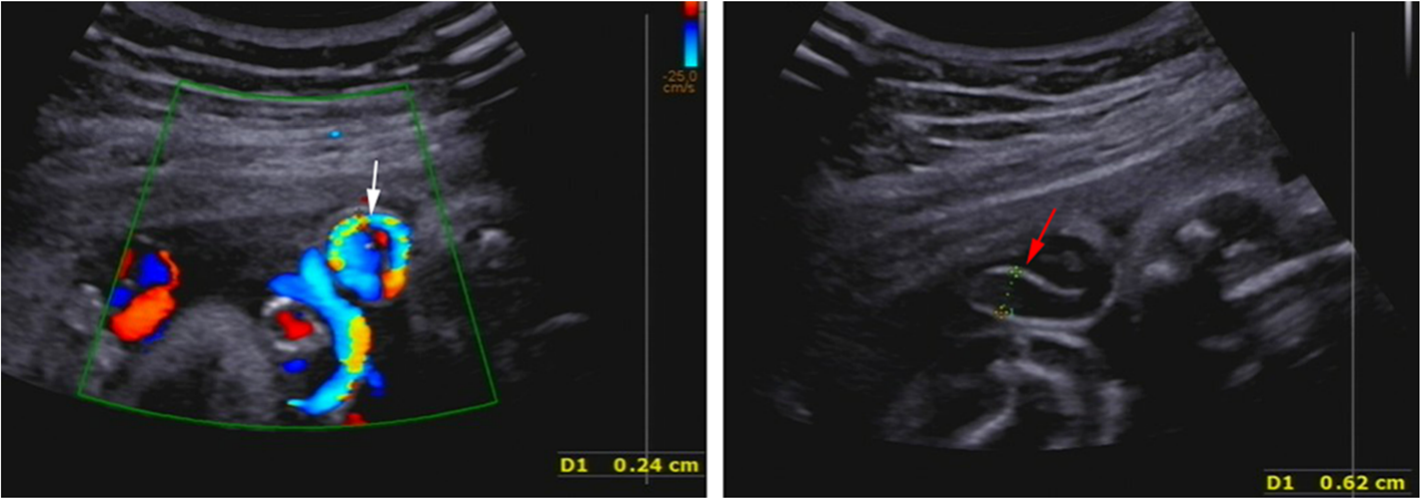
Ultrasonography images show stenosis (arrow) of the umbilical vein with a width of 0.24 cm, and color Doppler shows “aliasing” (an artifact that denotes high-speed flow) (left), while vein dilation (arrow) is observed near the section of stenosis (right). Both images were obtained at 31^+ 3^ weeks of gestation

The cytogenetic analysis by amniocentesis was normal at 30 weeks. FGR was evaluated by ultrasonography using the Hadlock formula owing to estimated fetal weight (EFW) of 1324 g (4.9th percentile), biparietal diameter (BPD) 7.14 cm (2.2th percentile), head circumference (HC) 26.87 cm (1.5th percentile), abdominal circumference (AC) 24.9 cm (8.9th percentile) and femur length (FL) 5.47 cm (3.9th percentile). The appearance of the umbilical venous walls was consistent with the prior ultrasound examination. Most of the umbilical cord was stacked into a mass (Fig. 1 right) but umbilical cord blood flow and amniotic fluid were within normal range. The patient described minor vaginal brownish bleeding, with irregular tightness in the lower abdomen, without other vaginal discharge, abdominal pain or uterine contraction at 31 weeks of gestation. The maternal white blood cell count (14.3 × 10^9^/L) was normal but serum CRP was increased at 12.6 mg/L. Fetal heart rate monitoring showed a spontaneous prolonged deceleration (Fig. 2 left). The result of the oxytocin challenge test (OCT) was positive for category II fetal heart rate tracings and late deceleration twice (Fig. 2 right). The biophysical profile (BPP) score was 8 of 10, which revealed an amniotic fluid index of 8.4 cm and fetal heart rate of 153 beats per minute. Two-dimensional ultrasound revealed that the umbilical venous walls were thickened and the local stenotic diameter of the umbilical vein was 0.24 cm with an area of vein dilation of 0.62 cm near the area of stenosis (Fig. 3 left, right). Color Doppler showed that the stenotic section of umbilical vein was observed to have local high-speed flow of 124.2 cm/s and decreased flow of 17.39 cm/s in the cord segments of vascular stenosis and near the fetal insertion, respectively, which was suggestive of local stenosis of the umbilical vein. A decreased umbilical artery peak systolic velocity (PSV) of 24.7 cm/s was obtained, along with a decreased umbilical artery pulsatility index (PI of 0.31), and a systolic-to-diastolic ratio (S/D) of 1.33. Doppler flow of the fetal middle cerebral artery and ductus venosus were normal. These findings suggested that the fetus was in a state of acute hypoxia but the brain had not yet been affected.

**Fig. 4:**
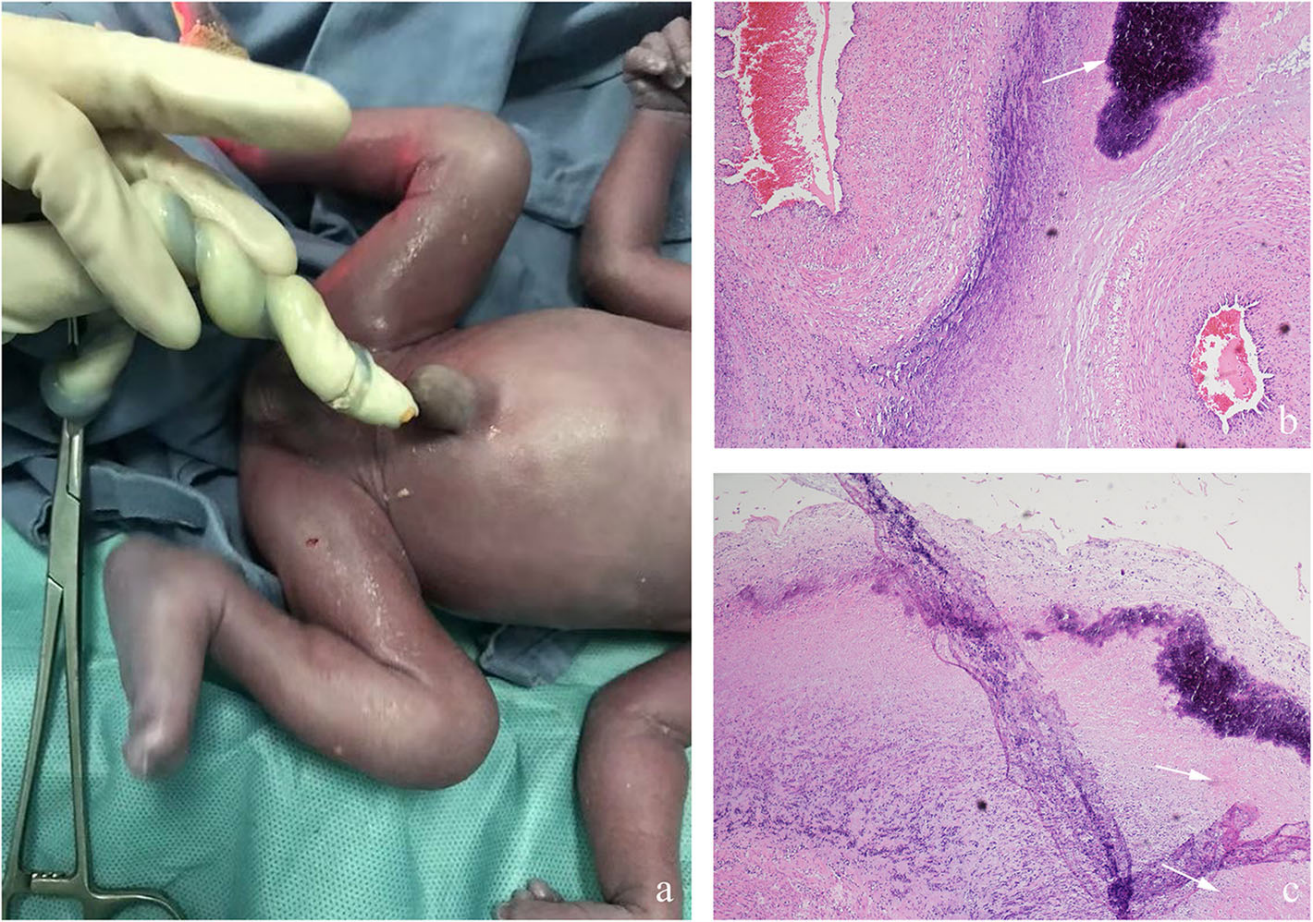
The color of fetal skin is dark and the umbilical cord is white, due to a white lipid-like substance within the umbilicus (**a**). The root of the umbilical cord ruptured during cesarean section. Histopathological examination of the placenta shows umbilical phlebitis and NF, revealing inflammatory cells (neutrophils and lymphocytes) as well as calcium (arrow) H&E, × 100 (**b**). Necrotic debris (arrow) is also seen H&E, × 100 (**c**)

Cesarean section was recommended due to evidence of fetal distress. Fortunately, a male infant was delivered in a timely manner. The baby was noted to have dark skin and a birth weight of 1390 g. Apgar scores were 8, 9 and 10 at 1, 5 and 10 min, respectively. Umbilical blood gas analysis showed a pH of 7.30, pCO_2_ of 51 mmHg, pO_2_ of 27 mmHg, and base excess (BE) of − 2.3 mmol/L. The fetal membranes were intact and the amniotic fluid was clear. Gross examination revealed that the umbilical cord had a white color from an internal white lipid-like substance, which was similar to but softer than typical calcification. The root of the umbilical cord was twisted and even fractured during the operation (Fig. 4 a). The pathological study demonstrated acute histological chorioamnionitis and NF (Fig. 4 b, c). The neonate had a work-up for sepsis. Results of placental and decidual cultures were negative.

The infant was transferred to a pediatric hospital for further treatment, and was admitted for preterm birth, acidosis, jaundice, respiratory distress syndrome (RDS), neonatal sepsis, very low birth weight (VLBW), small for gestational age (SGA) and electrolyte imbalance. Microbial examination results of TORCH, chlamydia pneumoniae and chlamydia trachomatis were negative. Microbial cultures of blood, urine and faeces were all negative. The baby had no fever. Sepsis was strongly suspected as the laboratory findings showed a white blood cell count of 20.4 × 10^9^ /L, a CRP of 16.0 mg/L and a Procalcitonin Test (PCT) of 4.46 ng/ml. There was no evidence of hepatomegaly and splenomegaly as demonstrated by ultrasonography. Imaging examinations found no brain lesion. The newborn was discharged in good clinical condition after 1 month of hospitalization that included antibiotic and supportive therapy. At the time of writing, the infant is currently 1 year old and healthy.

## Discussion and conclusions

Combing elevated maternal CRP, result of placental pathology and diagnosis of neonatal sepsis, intrauterine infection was considered highly likely in this case. The screenings for maternal and neonatal TORCH serology were negative. The microbial cultures of maternal placenta and decidua, and of neonatal blood, urine and faeces were all negative. Therefore, the causative organism of the presumed intrauterine infection was not identified. FGR was a resultant complication. Infection progressed to the umbilical cord and the fetal circulation, leading to NF and neonatal sepsis. Postnatal pathology confirmed that the calcification of the umbilical venous wall seen in ultrasonography was a manifestation of NF. Because of the umbilical cord torsion, blood flows of umbilical vein and umbilical arteries were abnormal. Prolonged deceleration of fetal heart rate was considered to be related to fetal hypoxia caused by umbilical cord torsion. Emergency cesarean section was performed due to fetal distress. Because of calcium deposition, the umbilical cord was brittle and even fractured during the operation. Without close monitoring and timely detection, this fetus would not have survived. Therefore, though the condition of NF is rare, it is important to pay attention to calcification of the umbilical vascular walls in prenatal ultrasonography.

There are few studies on NF and most articles were published a few decades ago. It is reported that the incidence of NF is 1.25% [3] and occurs in 0.1% of infants delivered at greater than 20 weeks of gestation [4]. The most common etiology of NF is intrauterine infection and most cases of NF have a polymicrobial etiology. Syphilis has been shown to be strongly associated with NF. Guarner et al. [5] found funisitis presenting in 42% and NF in 11% of mothers with syphilis. Other pathogenic infectious agents reported in association with NF include *Streptococcus pneumoniae*, herpes simplex virus, Actinomyces meyeri and vaginal yeast [4, 6–8]. Chorioamnionitis is regarded as a hallmark of maternal inflammation [9]. When inflammation progresses to the umbilical cord, a condition termed funisitis, this is considered to be a histopathologic hallmark of fetal inflammation response syndrome (FIRS) [10, 11]. Therefore, funisitis or even NF denotes the appearance of a fetal inflammation response.

NF is a distinctive inflammation of the umbilical cord, obvious both macroscopically and microscopically. On the macroscopic view, NF reveals yellowish-white chalky stripes paralleling the umbilical vessels, and it presents rings or crescents around blood vessels on cross-section. On the microscopic view, lesions include inflammatory cells, necrotic debris and calcium deposits [3]. Because cord tissue lacks capillaries, lymphatics and fixed macrophages [12], it is difficult for necrotic debris to be removed. Thus, necrotic inflammatory cells accumulate and gradually develop mineralization with deposition of calcium. This is the pathological mechanism by which NF presents with hyperechogenicity or calcification paralleling the umbilical vessel walls in ultrasonography. Through this mechanism, calcification occurs more frequently in longstanding NF. The incidence rate of cord calcification in NF is 47% (28 of 60) [4]. Jacques et al. [3] reported extensive calcification presenting in 6 of 45 cases, and the remaining 39 case demonstrated some degree of calcified material. Due to the deposition of necrotic debris and calcium, the umbilical cord becomes fragile, even progressing to intrapartum rupture of the umbilical cord, a severe complication of NF [2].

As in this case, many intrauterine infections are subclinical, making them difficult to detect. For pregnant woman with intrauterine infection or suspected intrauterine infection, the crucial point is whether there is fetal inflammation response. It is known that levels of biomarkers (such as IL-6 and CRP) in maternal blood can help diagnose intrauterine infection, but this involves complex immune regulatory mechanisms. It is not clear whether these biomarker levels can predict the severity of intrauterine infection [13, 14]. It is noted that in several NF cases diagnosed by postpartum pathology, prenatal fetal distress was found, related to an abnormal fetal heart rate [2, 15]. NF is prone to result in fetal distress, which will lead to intrauterine death if not found in time. The fetal heart rate monitoring patterns play an important role in assessing fetal risk during a pregnancy [16].

In ultrasonography examination, it is proven that left ventricular Tei index (an index of myocardial performance), left ventricular E/A ratio, the velocity time integral, small fetal thymus size, fetal adrenal gland volume and the splenic vein flow pattern play roles in diagnosing FIRS [17–21]. Additionally, the umbilical cord should be observed in prenatal ultrasonography. Katsura et al. [22] reported two cases of funisitis diagnosed by prenatal ultrasonography, which showed non-uniform thickening of Wharton’s jelly with heterogenous echoes, and a high echoic line of the umbilical vessel wall. Placental pathology showed the area of high echoic line was due to infiltration of inflammatory cells and plasma proteins. Previous reports have divided calcification of umbilical cord vessels into two types based on causation: thrombosis and intrauterine infection [1]. The former is characterized by complete obliteration of the lumen due to calcification occurring within the vessel. The latter type of calcification occurs in the umbilical vascular wall. Thus, the location of umbilical vessel calcification helps distinguish the two causative types.

In summary, NF is a rare and severe inflammation of the umbilical cord. The diagnosis of NF should be considered in the differential when calcification of umbilical vascular walls is discovered in prenatal ultrasonography, especially in pregnant women with suspected intrauterine infection. This is crucially important for both maternal counseling and infant management. Obstetric patients with such ultrasonographic findings require close observation, careful examination, more frequent clinical monitoring, fetal heart monitoring, laboratory examination and detailed ultrasonography. Treating physicians can exercise the best clinical judgement through such a comprehensive approach, in order to prevent adverse outcomes. Future documented cases of NF associated with umbilical vascular wall calcification seen on prenatal ultrasound will be valuable to publish. This would further substantiate the significance of the ultrasonographic findings in this case report.

## Supplementary Information

**Additional file 1.**

## Data Availability

All data analyzed during this study are included in this published article. The data and materials supplementary file does not contain any patient names.

## Abbreviations

NF: Necrotising funisitis
CRP: C-reactive protein
FGR: Fetal growth restriction
FIRS: Fetal inflammation response syndrome
