# Supplementary material for: Necrotizing funisitis and calcification of umbilical vein: case report and review
Source: BMC Pregnancy Childbirth. 2021 Apr 12;21:296. doi: 10.1186/s12884-021-03743-3 (PMC8042875; doi:10.1186/s12884-021-03743-3)
Supplement: Supplementary file 1 — Additional file 1. [file 12884_2021_3743_MOESM1_ESM.docx]

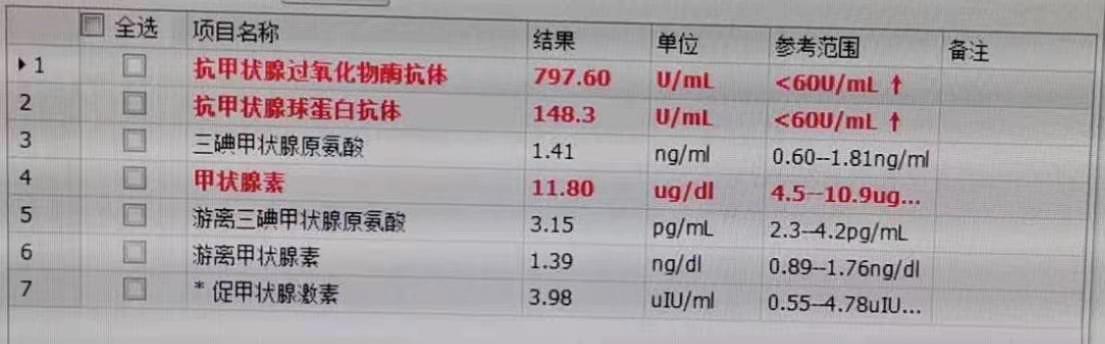
Thyroid function tests of the pregnant woman in first trimester.


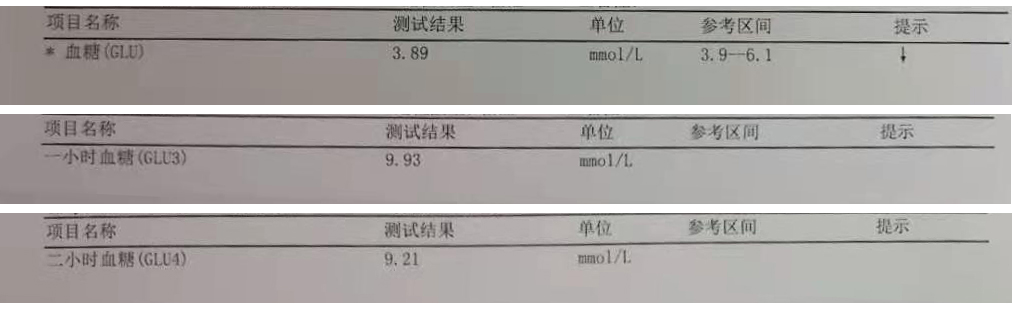


 75 g oral glucose tolerance test of the pregnant woman (fasting blood glucose-1 hour-2 hours)
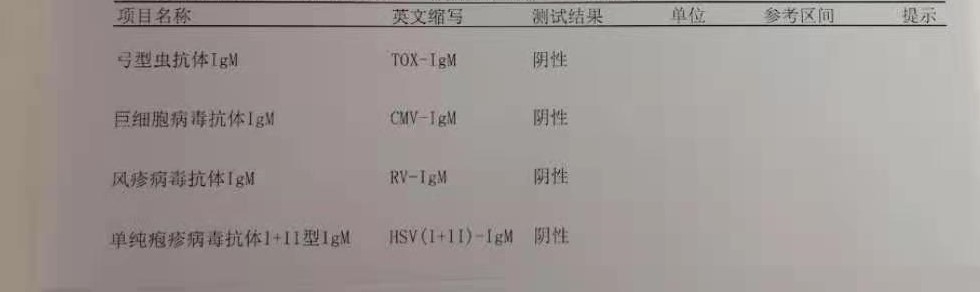


The screenings for TORCH of the pregnant woman at 25 weeks of gestation


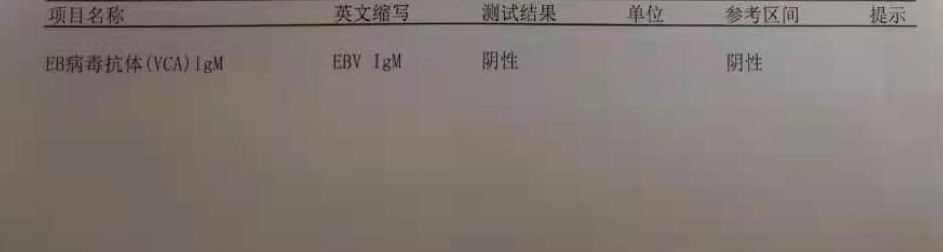


The screenings for epstein-barr virus of the pregnant woman at 25 weeks


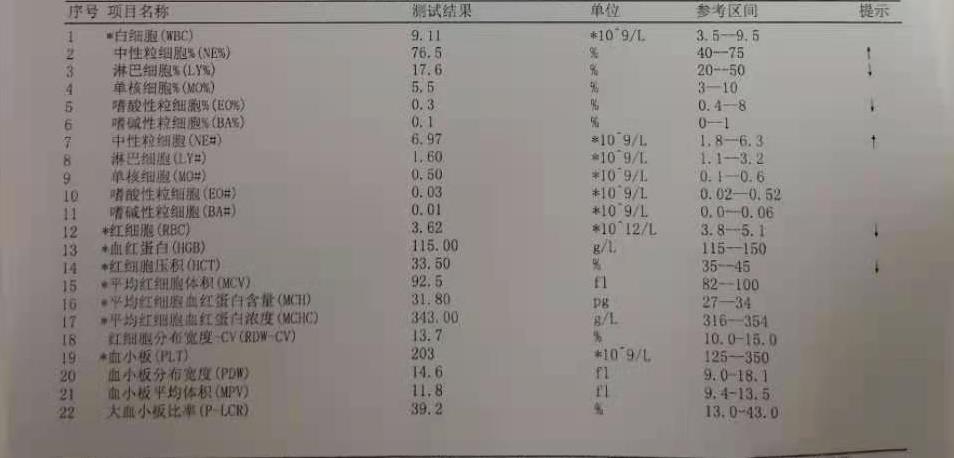


Routine blood examination of the pregnant woman at 26 weeks


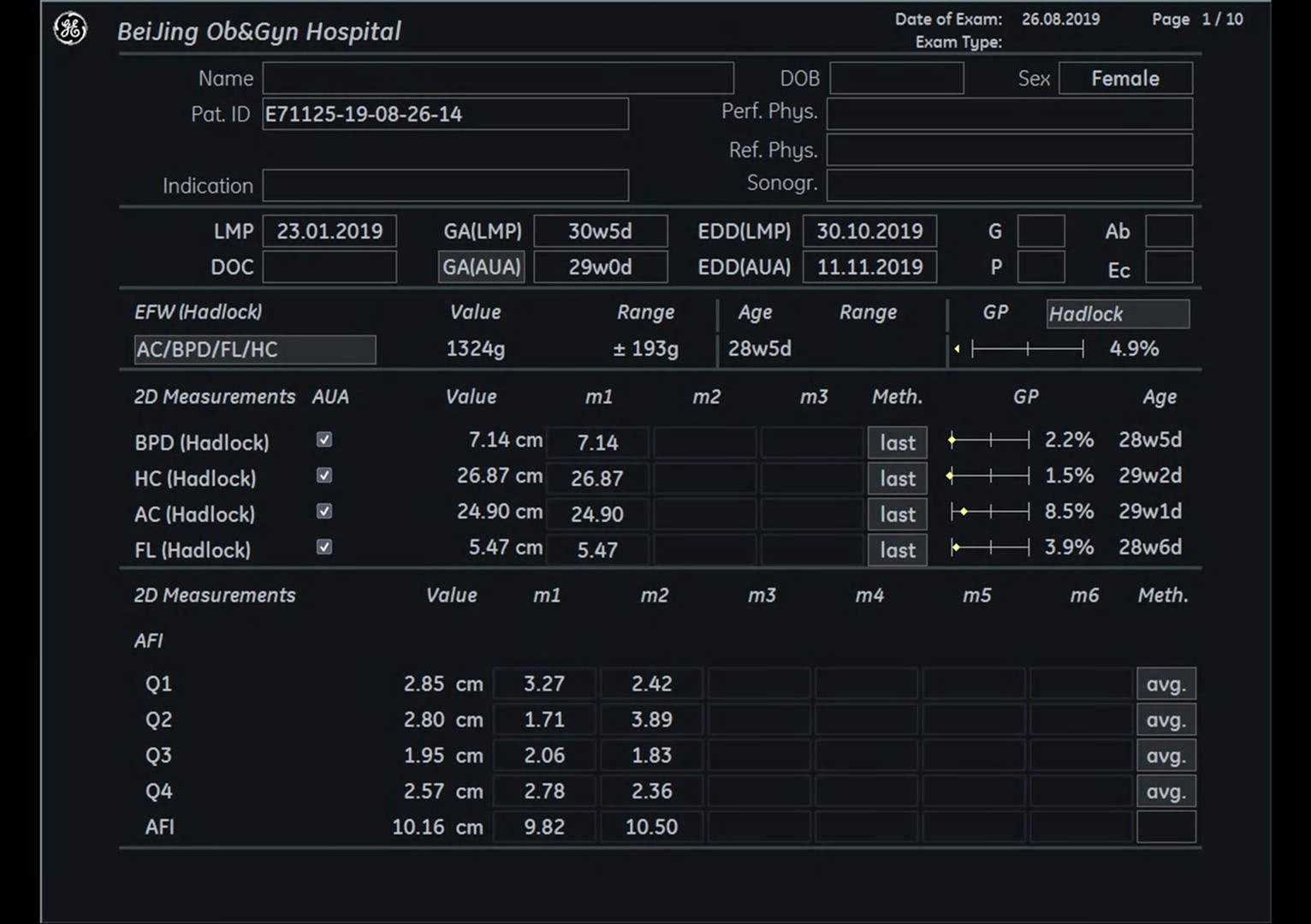


 Fetal growth parameters at 30 weeks


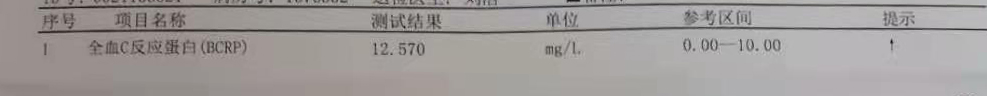


C-reactive protein of the pregnant woman at 31^+1^ weeks


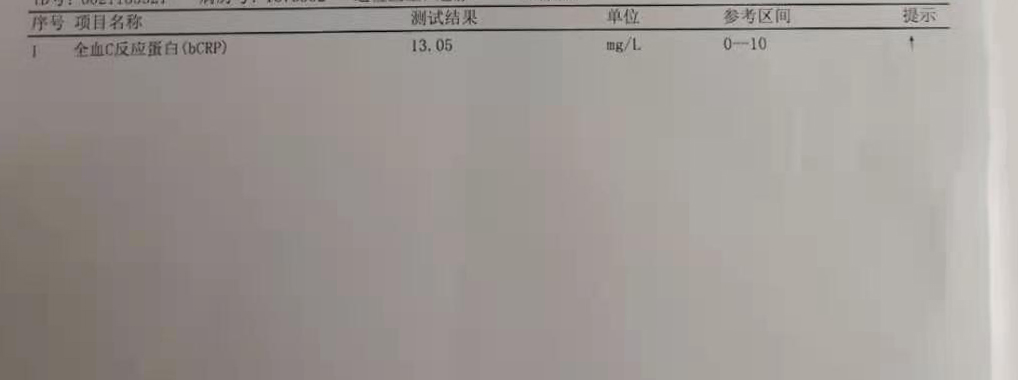


C-reactive protein of the pregnant woman at 31^+3^ weeks


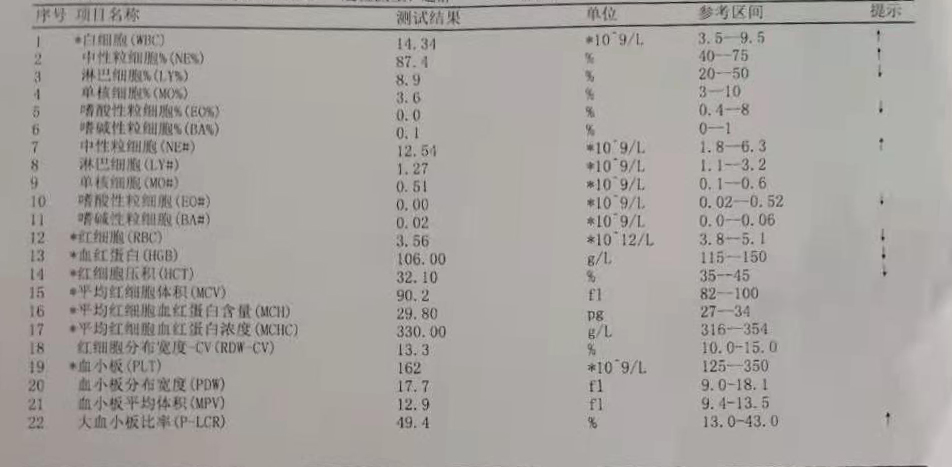


Routine blood examination of the pregnant woman at 31 weeks


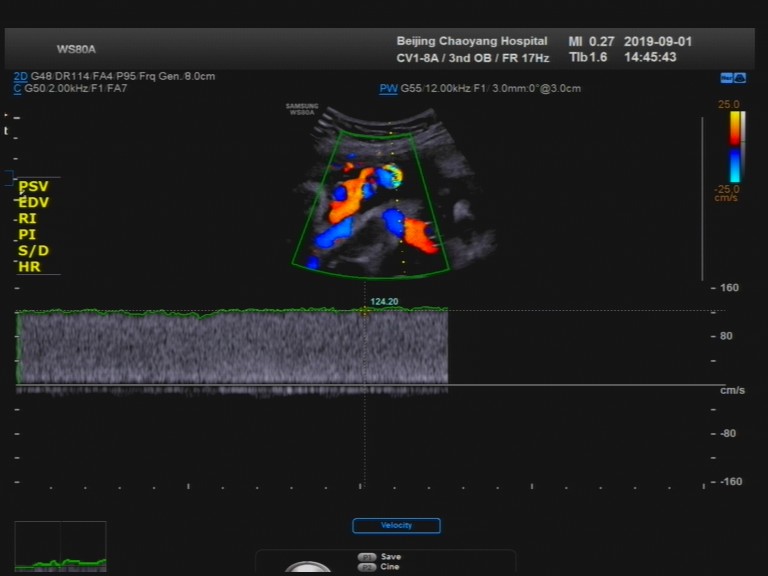


Color Doppler of fetal umbilical vein stenotic section at 31 weeks


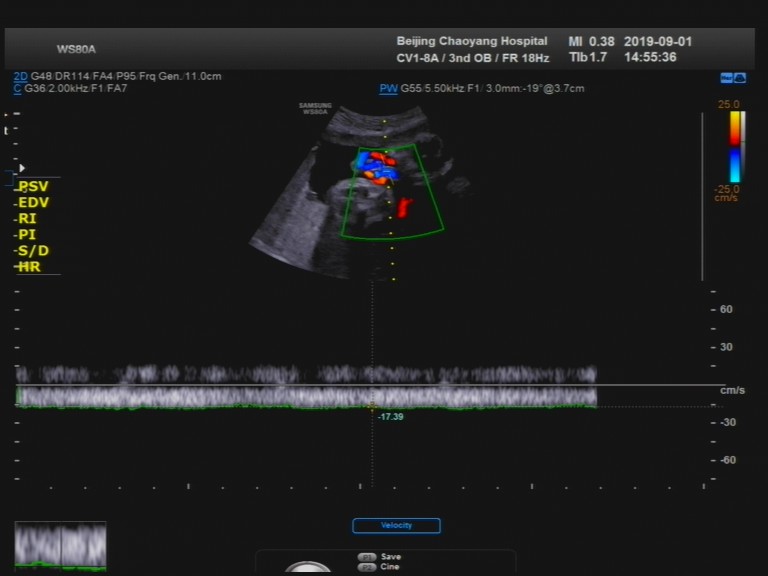


Color Doppler of fetal umbilical vein that near the area of stenosis section at 31 weeks


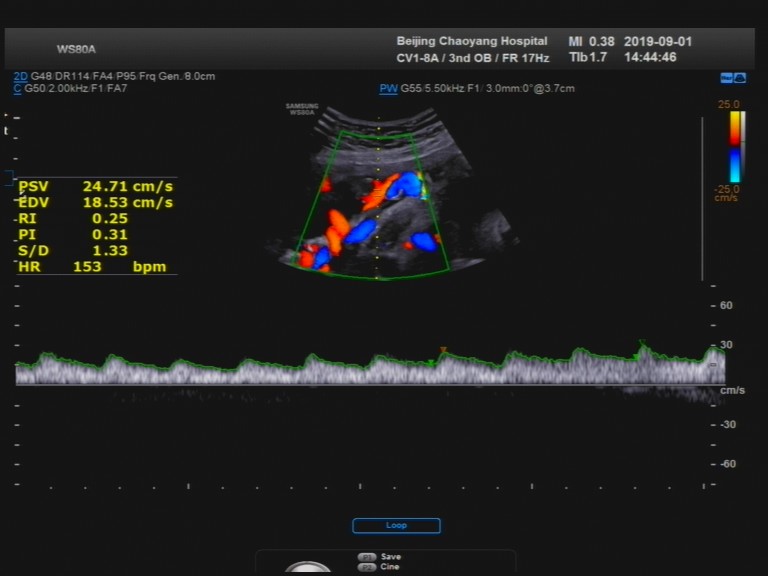


Color Doppler of fetal umbilical artery at 31 weeks


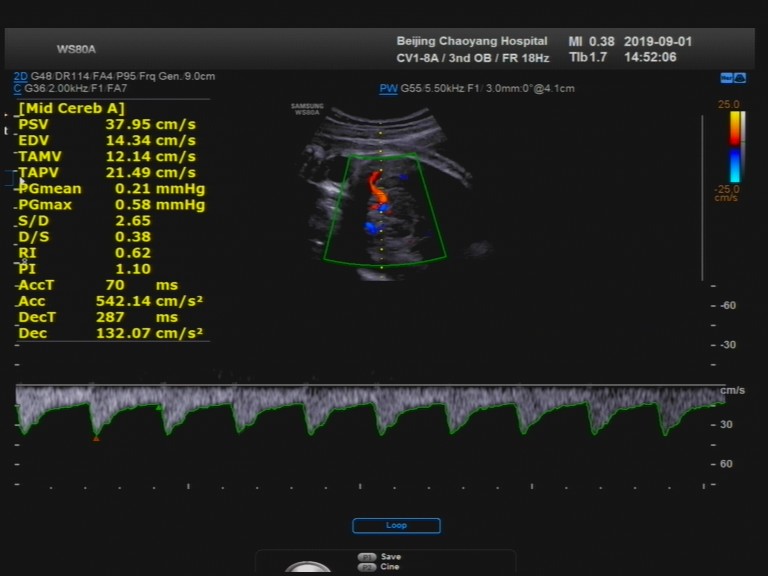


Color Doppler of fetal middle cerebral artery


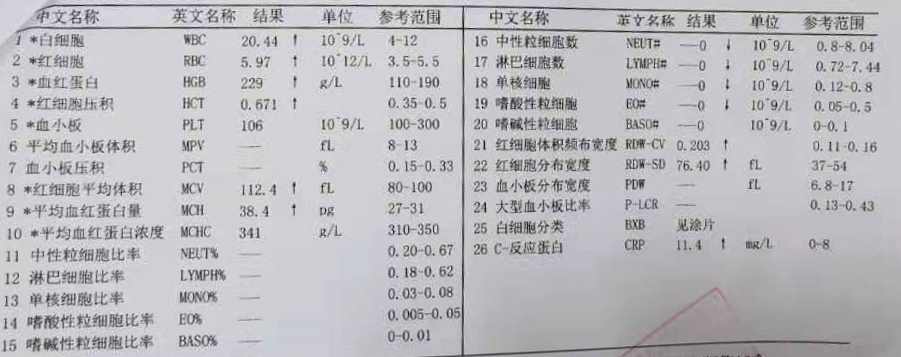


Routine blood of the infant 1 day old


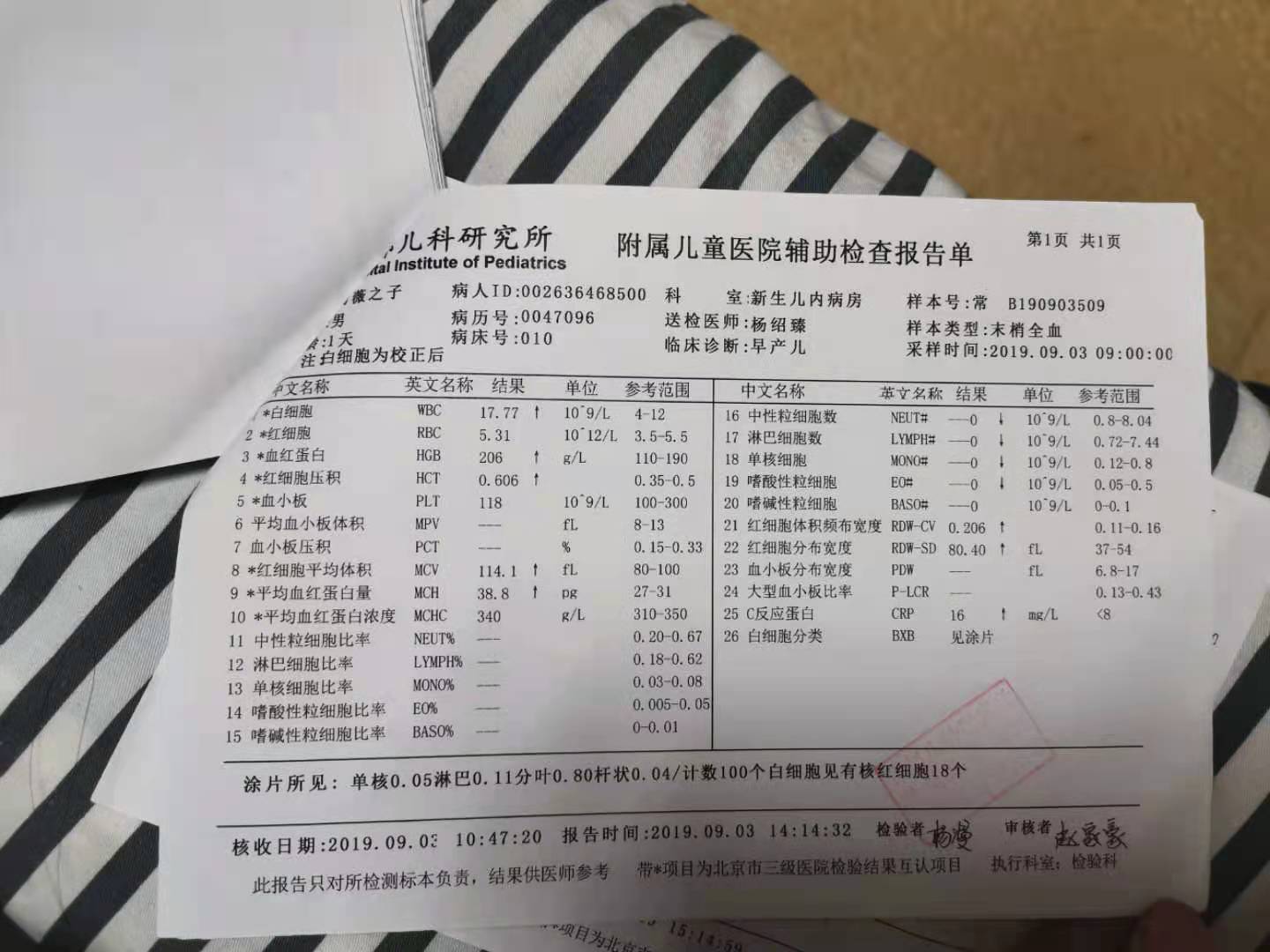


Routine blood of the infant 1 day old, too.


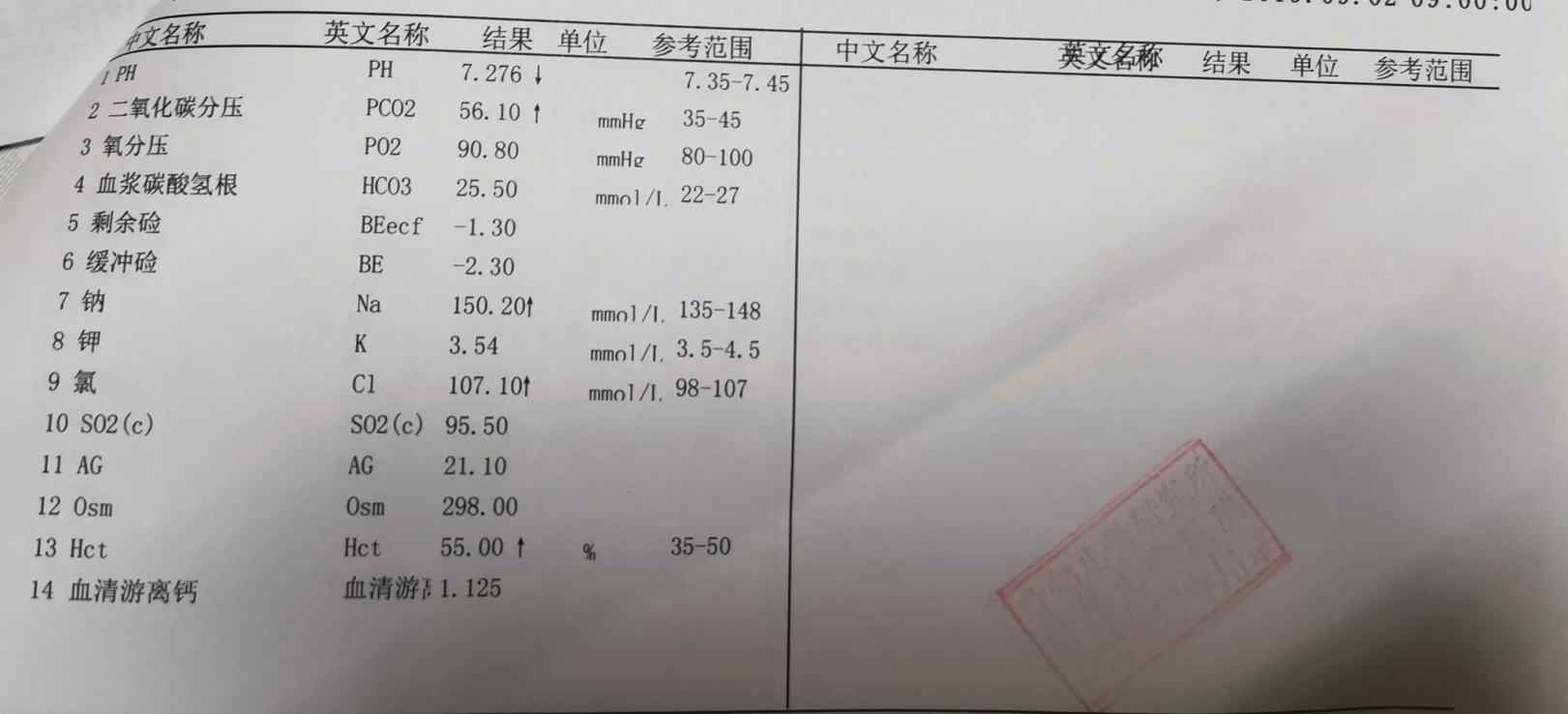


Arterial gas analysis of the infant 1 day old


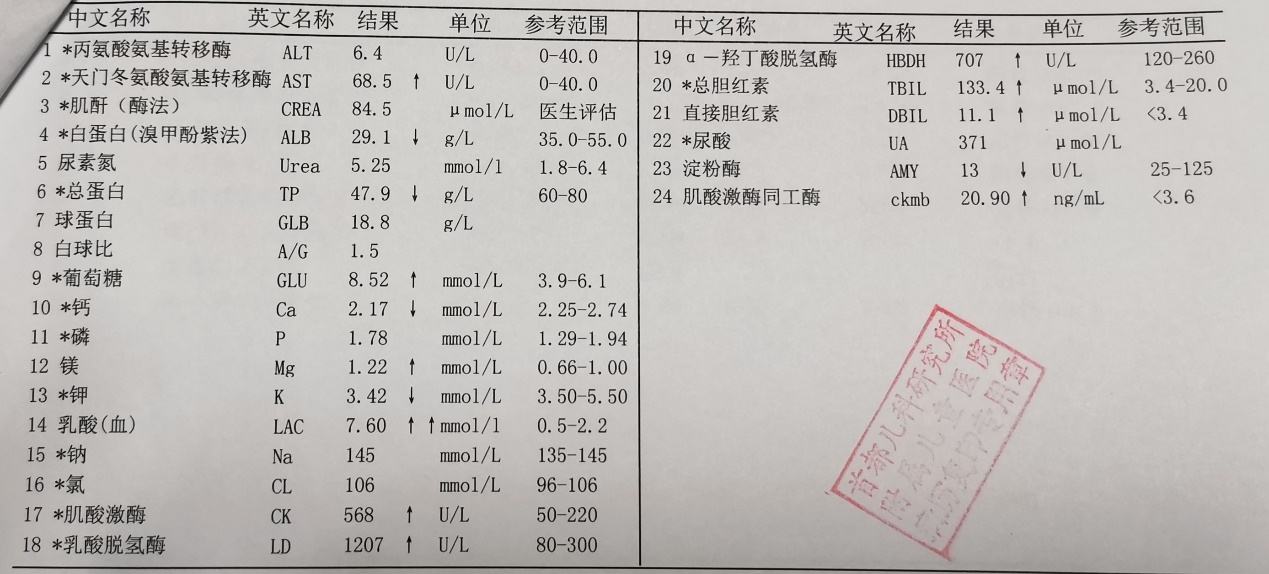


Laboratory data of the infant 1 day old


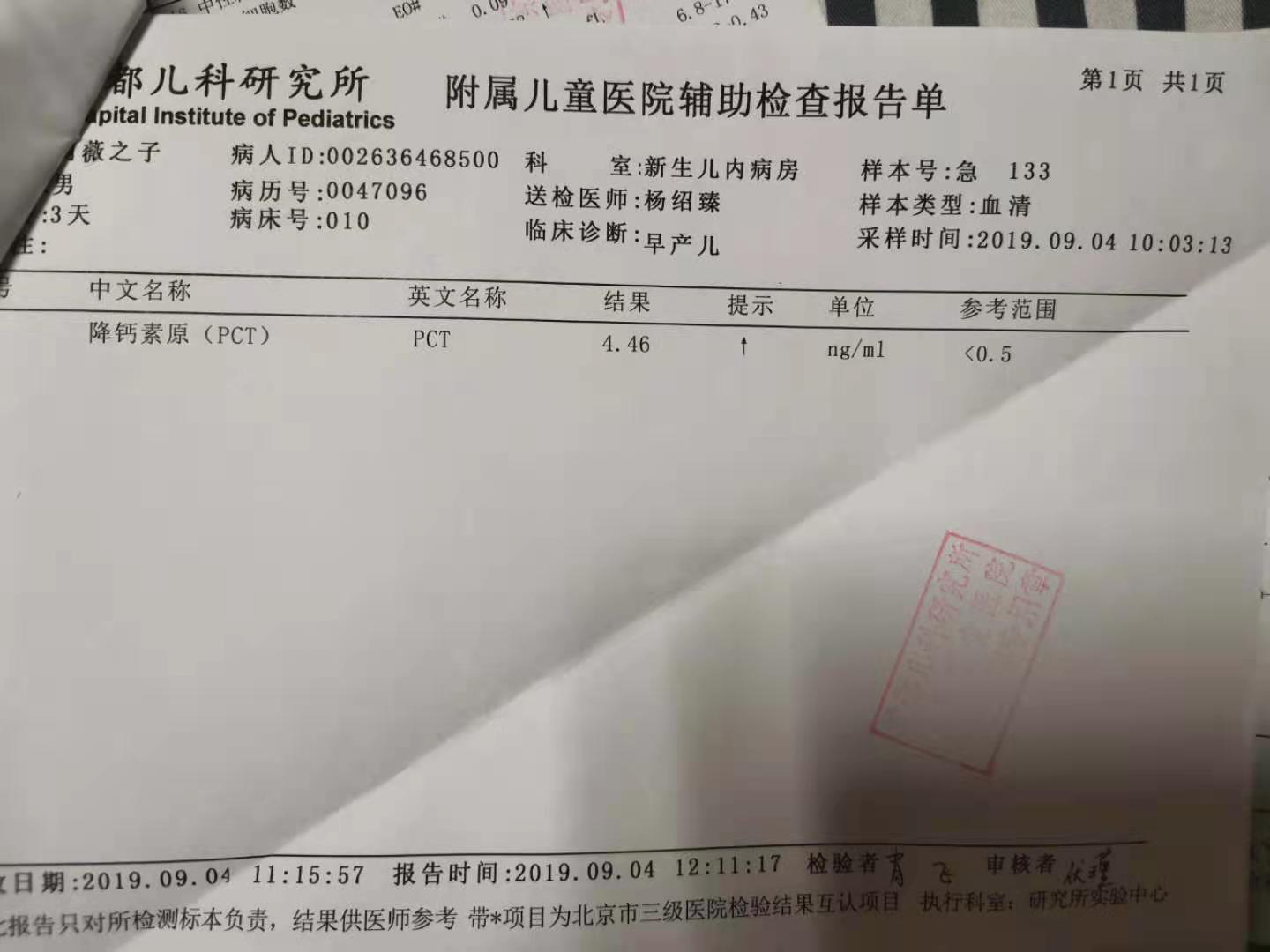
Procalcitonin Test of the infant 3 days old


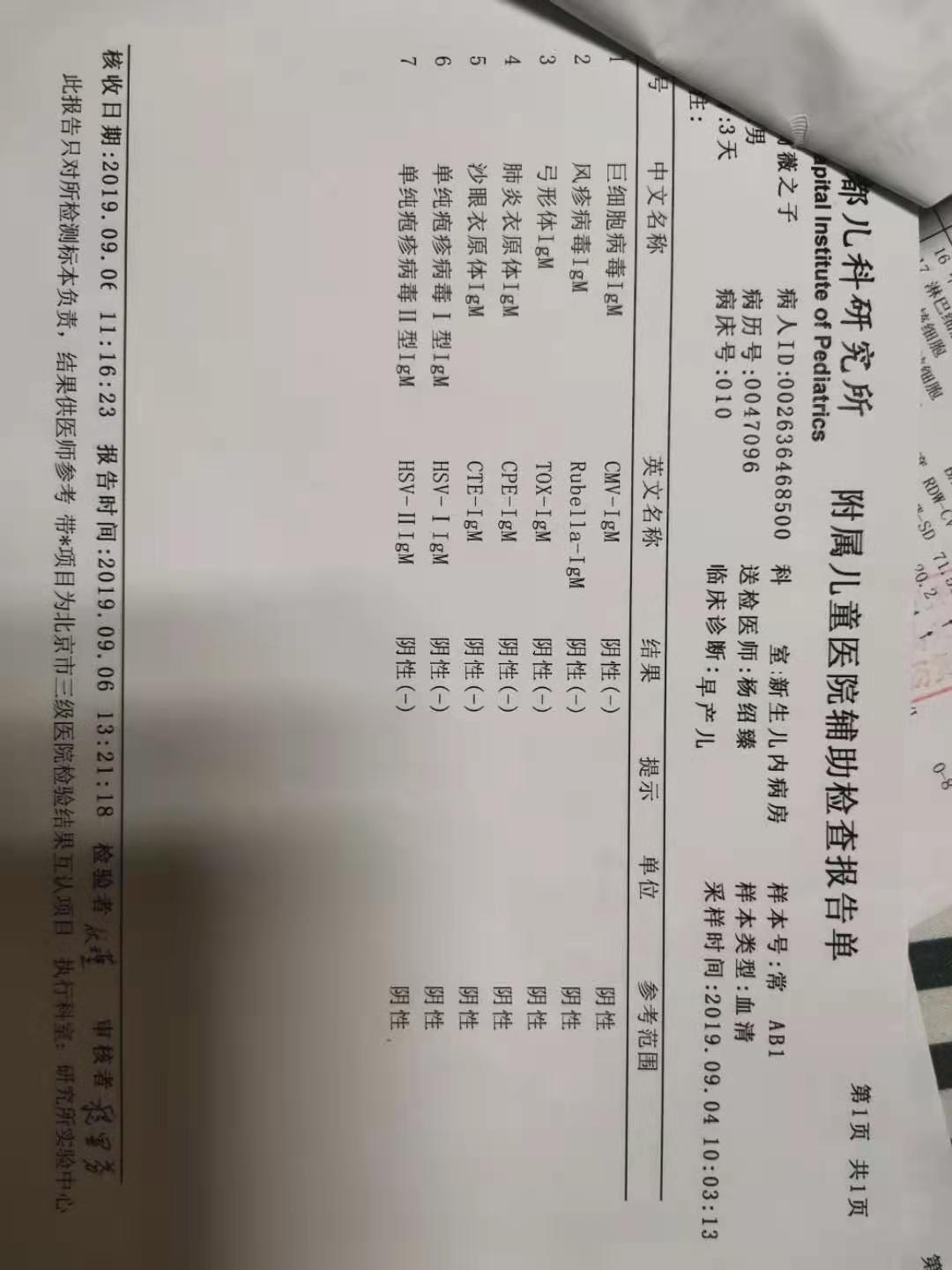


The screenings for TORCH of the infant 3 days old


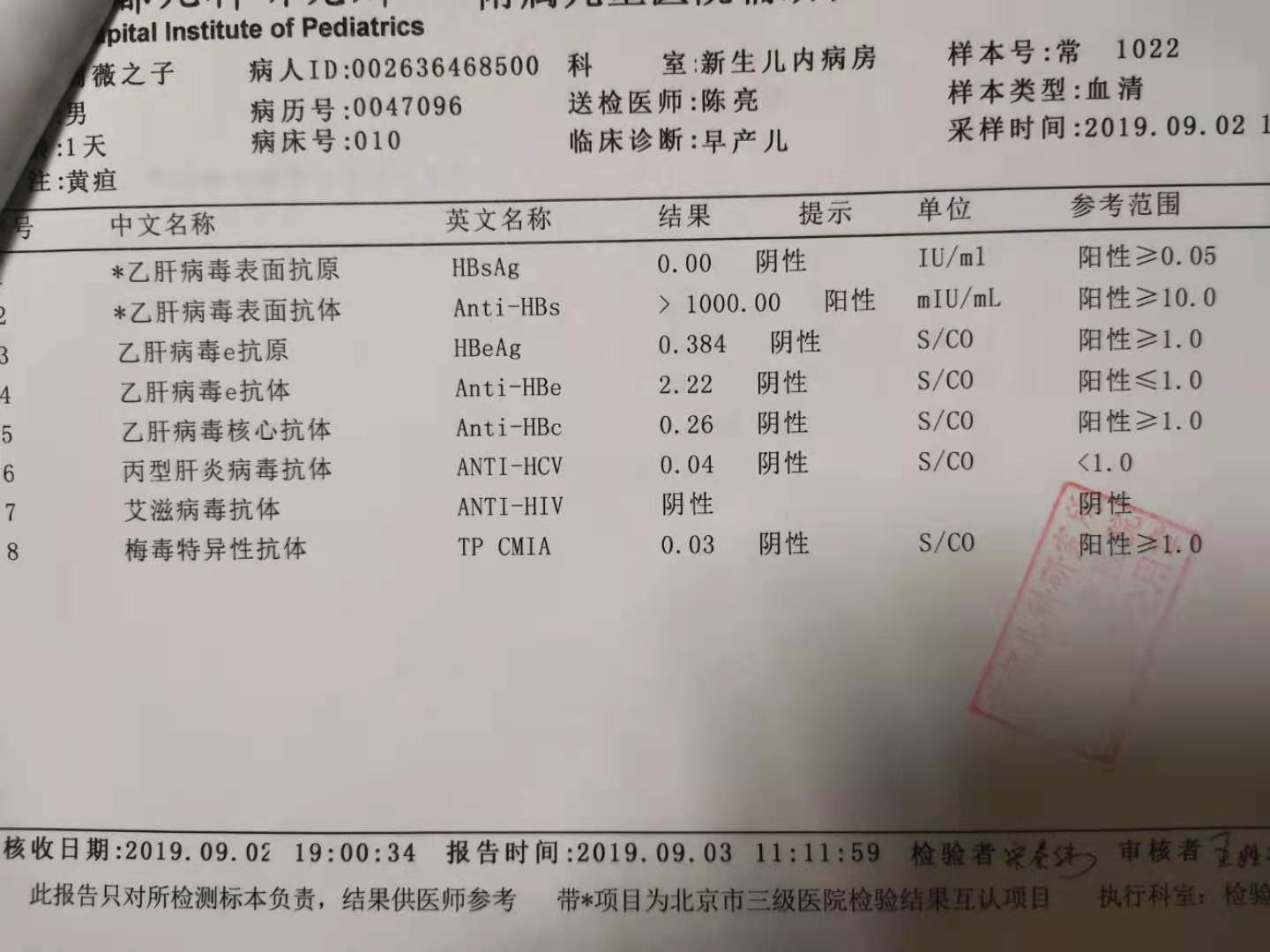


The screenings for infectious disease markers of the infant
